# Activation of endogenous retroviruses in tumor cells and their immunomodulatory mechanisms: from molecular basis to clinical translation

**DOI:** 10.3389/fonc.2026.1752231

**Published:** 2026-03-23

**Authors:** Jintong Zhang, Yanhua Zhang, Xiaochun Peng

**Affiliations:** 1 Laboratory of Oncology, Center for Molecular Medicine, School of Basic Medicine, Health Science Center, Yangtze University, Jingzhou, China; 2 Department of Physiology, School of Basic Medicine, Health Science Center, Yangtze University, Jingzhou, China

**Keywords:** biomarkers, endogenous retroviruses, epigenetic reprogramming, immune checkpoint inhibitors, tumor immune microenvironment, viral mimicry response

## Abstract

Endogenous retroviruses (ERVs), regarded as “molecular fossils” embedded within the human genome, have been shown to exhibit increasingly intimate associations with tumor initiation, progression, and immune evasion through aberrant activation events. This review aims to systematically dissect the molecular mechanisms underlying ERV reactivation in the tumor microenvironment (TME), which are mediated by epigenetic reprogramming, transcription factor network dysregulation, and genomic instability, while highlighting their dual role in immune modulation. On one hand, ERVs activate innate and adaptive antitumor immunity via “viral mimicry” responses; on the other hand, they can induce the expression of immune checkpoint molecules and foster an immunosuppressive TME, thereby facilitating tumor immune evasion. Leveraging recent advancements in single-cell multi-omics and spatial transcriptomics technologies, this review delineates the dynamic expression patterns of ERVs in tumor heterogeneity and integrates extensive preclinical and clinical trial data to illustrate the translational potential of ERV-targeted strategies in tumor diagnosis, prognostic assessment, and immunotherapy. Finally, this review proposes addressing current research bottlenecks by harnessing spatiotemporally precise gene-editing technologies and AI-driven ERV activity prediction models, thus offering a novel paradigm for the development of next-generation tumor immunotherapies.

## 1 Introduction

Endogenous retroviruses (ERVs) are genomic remnants left behind after ancient retroviruses infected host germ cells, integrating their viral genomes into the host genome in this way. This vertical transmission spanning millions of years has led to ERVs occupying a significant proportion of the human genome, approximately 8% of its total sequence (1). ERVs serve as key regulators of host physiology, playing a crucial role in a wide range of biological processes (2–4). However, abnormal activation of endogenous retroviruses has been closely associated with the occurrence and progression of various human malignancies, making endogenous retroviruses a paradoxical entity connecting evolutionary biology with modern clinical oncology (5). In the tumor microenvironment (TME), the originally tightly restricted silencing state of endogenous retroviruses is often disrupted. The hallmark features of cancer, such as widespread genomic instability, global epigenetic reprogramming, and persistent inflammatory signaling, collectively trigger extensive and abnormal reactivation of these dormant viral sequences (6). This leads to a series of immune regulatory outcomes with profound and intriguing dual properties. On the one hand, the re-expression of ERVs can have harmful effects on tumor cells. Antigens encoded by ERV open reading frames are recognized by the host immune system as “non-self” components. This generates a unique set of tumor-specific antigens capable of eliciting a strong anti-tumor immune response, providing new targets for clinical immunotherapy strategies (7). On the other hand, tumor cells can adapt and exploit this reactivation process. Chronic ERV activation may activate endogenous negative feedback regulatory circuits, including the induction of immune checkpoint molecules (such as PD-L1) and the infiltration of immunosuppressive cell populations. Overall, these alterations create an immune-tolerant environment, ultimately promoting tumor immune escape (8). It must be recognized that this complex immune regulatory function is part of a broader regulatory network. The regulation of immunosuppressive cell populations in the lung cancer microenvironment (such as myeloid-derived suppressor cells (MDSCs)) is not limited to ERVs. Other non-coding RNAs, especially long non-coding RNAs (lncRNAs), are also considered key upstream regulators of MDSC proliferation and functional execution (9, 10). These factors collectively weave a complex regulatory network that drives tumor immune suppression. This dynamic interaction highlights the necessity of dissecting ERV-mediated regulatory events within the comprehensive framework of the TME. ERVs have great potential for clinical translation, making them biomarkers for patient stratification prediction and new targets for combination therapeutic intervention (11). Despite these advances, it must be emphasized that most current research is still limited - typically confined to a single specific cancer type or focusing on only one or two ERV families (such as HERV-K or HERV-H). This limited focus leaves considerable gaps in our knowledge system, as we lack a systematic and global understanding of the comprehensive regulatory network of ERVs across the entire spectrum of human malignancies. This knowledge gap constitutes the key entry point and main rationale for this study (12). Although previous studies have begun to map the activity of ERVs in tumors, existing results still have many limitations (13). Firstly, the heavy reliance on large-scale sequencing technologies, such as high-throughput RNA sequencing, results in the averaging of signals from millions of cells, thus impeding the analysis of the profound heterogeneity of ERV expression. We are unable to distinguish between ERV expression patterns within malignant cells and adjacent immune cells, nor can we interpret the crucial interactions between them (14). Secondly, there is a notable lack of in-depth mechanistic research. We have yet to fully elucidate the role of ERVs as “molecular bridges,” how they integrate upstream carcinogenic signals (such as metabolic stress or oncogene activation) and transform them into precise regulation of downstream immune responses (15). Furthermore, translating the findings of basic research into clinically feasible and robust diagnostic strategies, or well-tolerated and effective treatment methods, still faces significant and multifaceted challenges (16). This review will focus on the latest research findings regarding human endogenous retroviruses (HERVs), emphasizing their unique aspects of activation and expression in tumors, with a particular focus on their implications for tumor immunotherapy (17).

## 2 ERVs and cancer

HERVs is derived from the exogenous retrovirus of our ancestors, and its genetic material has been integrated into our germ line DNA. The current classification of HERV depends on the initiation of reverse transcription by amino acids coupled with tRNA binding to viral primer binding sites (PBS). For example, Members of the HERV-K family use lysine (k) tRNA to initiate reverse transcription. HERV is currently divided into three categories: Class I including HERV -H, HERV -F, HERV-W,HERV-R, HERV-P, HERV-E,HERV-I, HERV-T, ERV-FTD,ERV-FRD; Class II including HERV-K ; Class III 3 including HERV-L (18). Among the known HERV family, HERV-K is recently obtained and further divided into 11 subgroups (HML-1 – HML-11). New HERVs and further groupings will be found more and more.

In particular, preliminary evidence shows that the etiological cofactors of HERV play a role in cancer development by stimulating cell fusion and immunosuppression of env protein. However, more and more evidence shows that HERV has a protective effect in some tumors. These comparative observations highlight the complex aspects of HERV activation in human diseases, especially cancer. Studies have shown HERV play the role during many kinds of cancer and also as targets of immunotherapy (19–21).

## 3 Mechanism of ERVs reactivation

**Figure 1:**
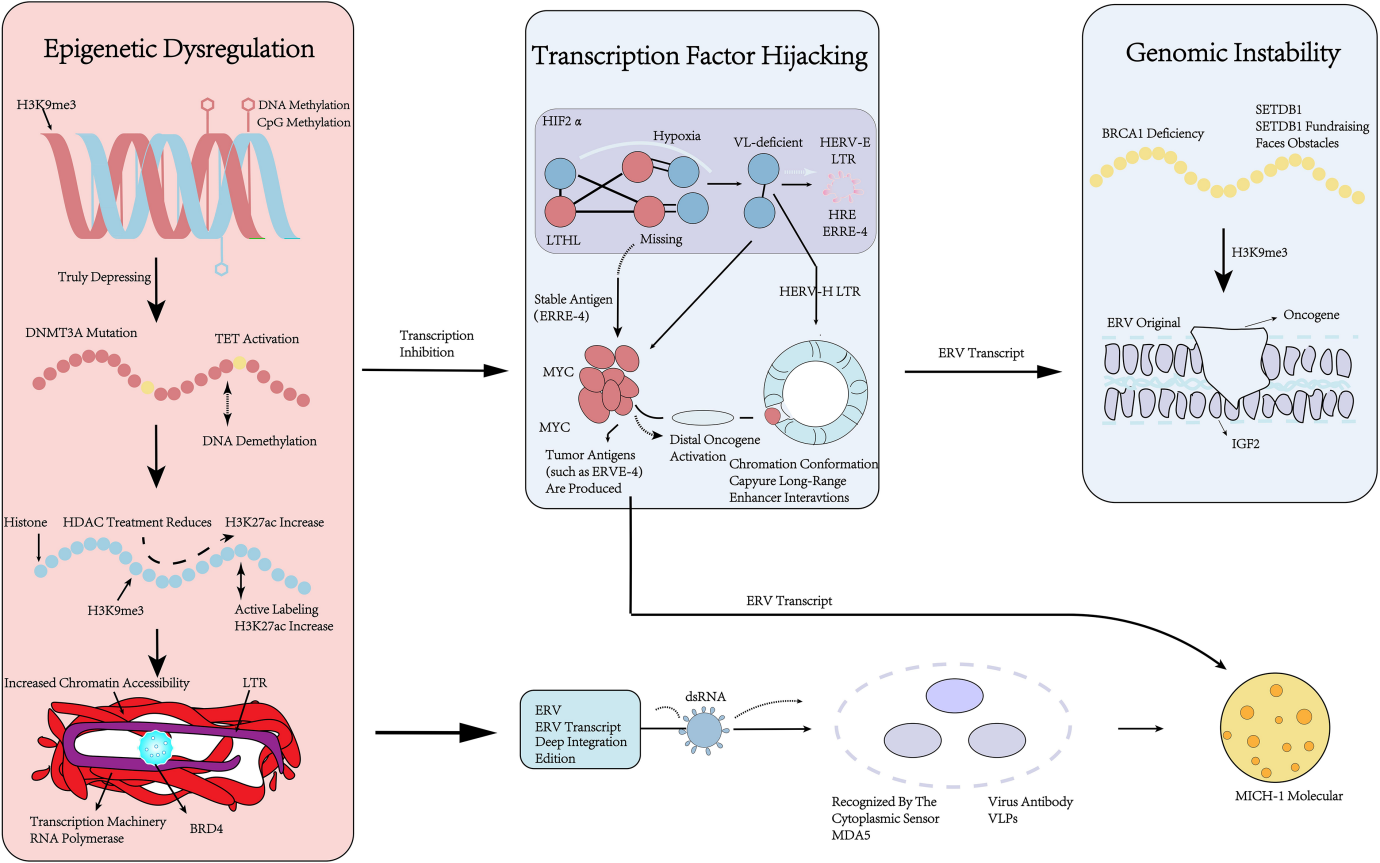
Schematic diagram of the three core mechanisms for ERVs reactivation in cancer.

The reactivation of ERVs in tumors is a crucial stage driving enhanced immunosuppression, which in turn promotes tumor invasion. This process is mediated by three core mechanisms: epigenetic dysregulation, transcription factor hijacking, and genomic instability (**Figure 1**).

### 3.1 Dynamic imbalance of epigenetic regulatory network

Epigenetic silencing is the primary and most robust mechanism by which host cells regulate the expression of ERVs. This process is crucial for maintaining genomic stability and preventing insertional mutations or aberrant transcription. However, in the complex process of tumorigenesis, this delicately balanced regulatory network is often severely disrupted. The collapse of epigenetic control is the primary driving force that unleashes the potential transcriptional potential of ERVs (22).

DNA methylation serves as a physical barrier, directly blocking the binding of transcription factors and recruiting methyl-CpG binding domain (MBD) proteins, which in turn assemble into larger co-repressive complexes. Studies on colorectal cancer organoid models have found that the CRISPR-dCas9-TET1 system targets HERV-K long terminal repeat (LTR) regions for demethylation, observing a significant upregulation of HERV-K transcription levels. Subsequent immunoelectron microscopy observations visually confirmed the accumulation of cytoplasmic double-stranded RNA (dsRNA) and the consequent functional impact of activating the antiviral MDA5/MAVS pathway (23). Consistent with this finding, analysis of The Cancer Genome Atlas (TCGA) pan-cancer data reveals that patients with acute myeloid leukemia (AML) carrying mutations in the *de novo* methyltransferase DNMT3A exhibit a significant increase in HERV-E expression. This epigenetic dysfunction is directly and significantly associated with poor patient prognosis, thus establishing a clear link between methylation mechanism defects, HERV reactivation, and clinical outcomes (24). Histone modification is also crucial in regulating the transcriptional accessibility of ERV loci. Researchers have successfully mapped the high-resolution, high-precision profiles of ERV site-specific H3K27ac (an activation mark associated with active enhancers and promoters) and H3K9me3 (a heterochromatin-type repressive mark) in breast cancer cells using advanced epigenetic analysis technology, chromatin endonuclease cleavage and tagging (CUT&Tag) (25). After treatment with histone deacetylase inhibitor (HDACi) SAHA, an acetyltransferase inhibitor, the signal intensity of H3K27ac in the HERV-H long terminal repeats (LTRs) region increased dramatically (26, 27). This subsequently recruited the transcription machinery, triggering a strong and sustained burst of transcription from HERV-H elements (28, 29). Notably, there is extensive and complex interaction between histone modification and DNA methylation in the regulation of ERV expression (30).

### 3.2 Reprogramming of transcription factor networks

Abnormal overexpression or mutational activation of specific transcription factors (TFs) in tumor cells, often as a direct consequence of carcinogenic driver events, may lead to the “hijacking” of ERV regulatory elements (**Table 1**). These transcription factors can directly bind to LTRs, converting these dormant viral sequences into new enhancers or promoters. These hijacked regulatory elements then strongly drive the expression of ERVs themselves, as well as adjacent oncogenes in some cases, thereby making ERVs a tool for promoting tumor progression (31).

**Table 1:** Key experimental evidence for transcription factors regulating ERVs activation.

Transcription factor	Binding site	Main verification technologies	Tumor type	Main effect	References
HIF2α	HERV-E LTR	ChIP-seq, fluorescent reporter gene	Renal clear cell carcinoma	Transcriptional activation increases by 12-fold, leading to the production of tumor antigens	(5, 33, 34)
MYC	HERV-H LTR	ATAC-seq, 3C chromatin conformation capture	Lymphoma	Chromatin accessibility is increased by 6 times, and long-range interactions are enhanced	(37, 38)
Mutant p53	HERV-K LTR	CUT&RUN, CRISPRa	Renal clear cell carcinoma	Increased both proliferation and invasion	(40)
NF-κB p65	HERV-W env	EMSA, luciferase reporter gene	Liver cancer	Promote the secretion of inflammatory factors and recruit immune cells	(41)
BRD4	HERV-K/HERV-H	BET inhibitor (JQ1), ChIP-seq	Leukemia, various solid tumors	Driving HERV transcription through super-enhancers to maintain a carcinogenic state	(29)
SETDB1 (deficiency)	HERV-K	CRISPRfilter, ChIP-qPCR	BRCA1-deficient breast cancer	The absence leads to the loss of H3K9me3 modification, resulting in HERV-K activation	(43, 44)

The VHL protein is a crucial component of the E3 ubiquitin ligase complex, which targets hypoxia-inducible factors (HIFs) for proteasomal degradation under normoxic conditions. Its inactivation leads to the constitutive stabilization and accumulation of HIF2α, even at normal oxygen levels (32). In-depth mechanistic studies have revealed that this stabilized HIF2α can directly bind to the LTR enhancer region of the HERV-E family (33). To functionally validate this binding event, researchers constructed an experimental system using GFP reporter genes driven by HERV-E LTRs. These experiments confirmed that under hypoxic conditions or only after HIF2α overexpression, the intensity of the fluorescent signal significantly increased, demonstrating that HIF2α binding is sufficient to drive the transcription of these LTRs (34). More importantly, from a therapeutic perspective, subsequent mass spectrometry analysis of the clear cell renal cell carcinoma (ccRCC) immunopeptidome identified a specific HLA-A11-restricted antigenic peptide CT-RCC-1, which is encoded by the env gene of this reactivated HERV-E. This discovery provides a specific and clear candidate target for the development of subsequent immunotherapies, such as cancer vaccines or T-cell-based therapies (35, 36). The oncogene MYC is a major regulator of cell proliferation, exhibiting significant overactivity in various human malignancies, including various lymphomas. Its role in the regulation of ERVs is an emerging research area. Researchers utilized transposase-accessible chromatin sequencing assay for Transposase-Accessible Chromatin with sequencing (ATAC-seq) technology revealed that forced overexpression of MYC can significantly reshape the chromatin landscape, leading to a marked increase in chromatin accessibility at HERV-H loci (37). These experimental results are astonishing, confirming that MYC can serve as a molecular mediator or “scaffold” to facilitate *de novo* physical interactions between distal HERV-H LTRs and enhancers of established oncogenes, such as Insulin-like Growth Factor 2 (IGF2), at a distance. By promoting the formation of these chromatin loops, MYC effectively expands the entire carcinogenic transcriptional program (38). This complex mechanism unveils a broader and more covert role of ERVs: they may serve as key long-range regulatory elements or “enhancer platforms” utilized by oncogenes to directly participate in the initiation and maintenance of tumor states (39).

### 3.3 Cascade effects of genomic instability

The inherent genomic instability of tumor cells represents another critical and unique pathway leading to the catastrophic collapse of ERV silencing mechanisms (42). This is particularly evident in breast cancer models with defects in the BRCA1 gene, a key tumor suppressor involved in DNA repair. Through a genome-wide CRISPR screening approach, the histone H3K9 trimethyltransferase SETDB1 was identified as a crucial “caretaker” factor, essential for maintaining the silencing and heterochromatin status of ERVs (43). Subsequent mechanistic studies revealed precise molecular connections: the absence of functional BRCA1 protein severely impairs the correct recruitment of SETDB1 to ERV loci. This recruitment failure directly leads to a significant local loss of the inhibitory histone mark H3K9me3 (44). Subsequently, using high-resolution, single-molecule RNA fluorescence *in situ* hybridization (smFISH) technology, researchers were able to visually track the fate of individual ERV transcripts. They observed a significant increase in the nucleocytoplasmic transport of HERV-K RNA, confirming the successful transcription and processing of these viral elements. This molecular cascade ultimately leads to the *de novo* activation of HERV-K and the subsequent formation of fully assembled virus-like particles (VLPs) in the cytoplasm (45). Furthermore, in hematological malignancies such as leukemia, large-scale chromosomal translocation events can physically relocate ERV elements (such as those from the ERV9 family) from their normally sequestered genomic locations to new, transcriptionally active chromatin regions (46).

## 4 The regulatory effect of ERVs reactivation on tumor immunity

**Figure 2:**
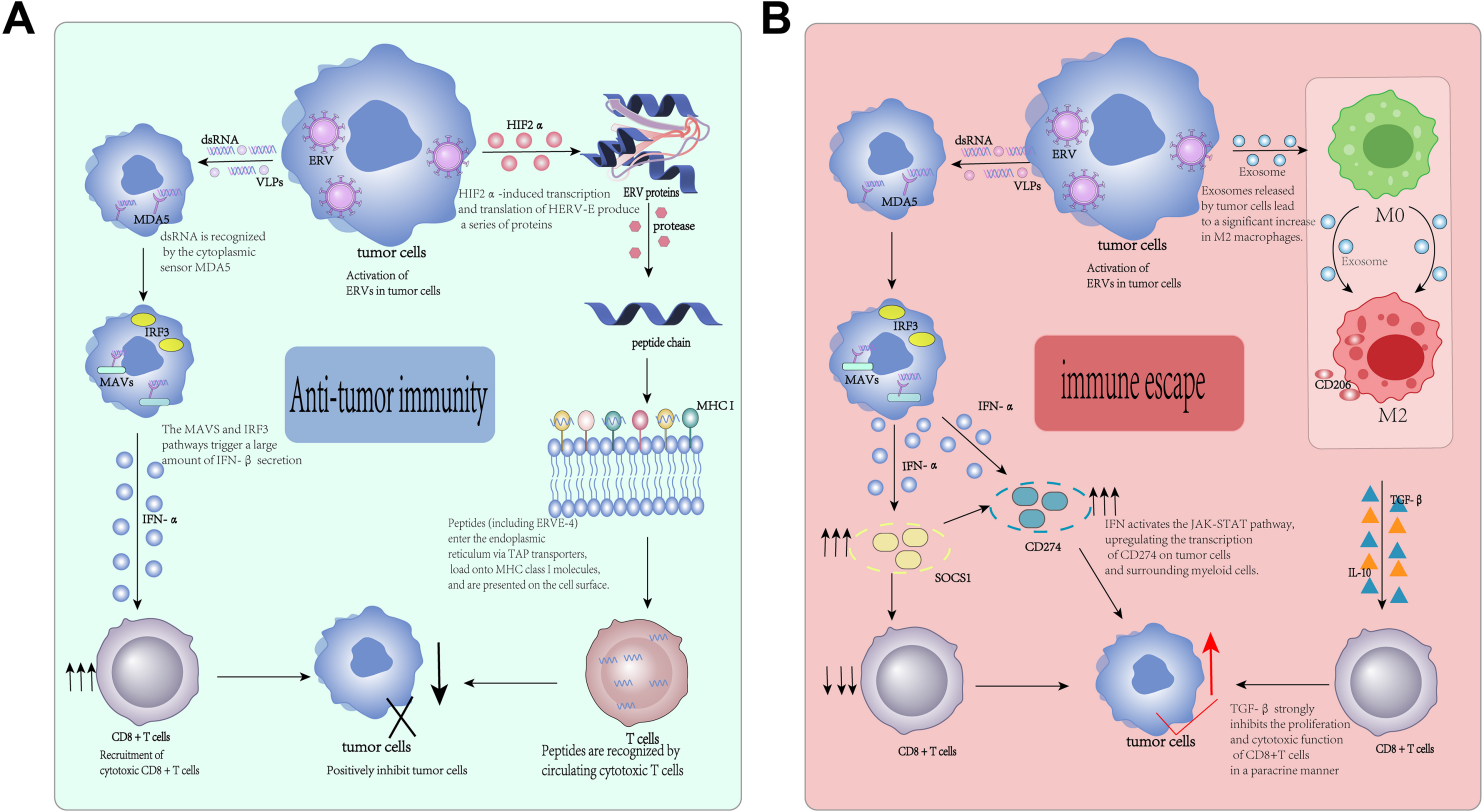
Dual immunomodulatory effects of reactivated ERVs in the TME. **(A)** Anti-tumor immune response induced by ERVs: ERV-derived dsRNA activates the IFN pathway to recruit CD8+ T cells, while ERV peptides presented by MHC-I trigger specific T cell killing. **(B)** Immune escape mechanism: chronic IFN signaling upregulates PD-L1, and ERV-containing exosomes polarize macrophages to M2 type, collectively inhibiting CD8+ T cell function. (II) Regulation of adaptive immunity: from antigen presentation to T cell response.

The reactivation of ERVs is by no means an isolated, cell-intrinsic event. Instead, it constitutes a profound biological process that fundamentally reshapes the entire TME (47). Among its various downstream consequences, aberrant ERV expression - by directly or indirectly regulating the function, polarization, and recruitment of key immune cells - emerges as one of the core factors determining the overall immune status of the TME (such as “hot” versus “cold”) (**Figure 2**) (48).

The transcription products of ERVs, especially the double-stranded RNA (dsRNA) intermediates, and in some cases fully formed virus-like particles (VLPs) - are molecular patterns commonly associated with exogenous viral infections. When these products accumulate in the cytoplasm, they are recognized as “danger signals” by host pattern recognition receptors (PRRs). This recognition triggers a series of antiviral signaling cascades, effectively mimicking viral infection and thereby activating the innate immune system. This phenomenon is aptly termed “viral mimicry” (49, 50). Of course, as antigens, they can also activate tumor immunity. In a pancreatic cancer model engineered to lack MED12, this pathway has been dissected in detail. In this experimental system, researchers observed a clear linear activation cascade: MED12 deficiency leads to abnormal nuclear localization of heterochromatin protein HP1α. This mislocalization, in turn, results in a widespread reduction of H3K9me3 modification specific to ERV loci, ultimately leading to transcriptional activation of HERV-K (51). Electron microscopy revealed that the functional consequence of this activation is the *de novo* formation of VLPs with a diameter of approximately 80 nanometers within tumor cells (52). The double-stranded RNA (dsRNA) produced during this process is recognized by the cytoplasmic sensor MDA5, followed by critical mitochondrial antiviral signaling The (MAVS) protein and the transcription factor IRF3 pathway trigger the massive secretion of interferon-β (IFN-β) (53). This innate immune activation has profound effects on the adaptive immune system. Subsequent flow cytometry analysis of tumor-infiltrating cells revealed an increase in the number of cytotoxic CD8^+^ T cells in the TME (54).To emphasize the clinical significance of this mechanism, a retrospective survival analysis of human pancreatic cancer patients further confirmed that patients with lower tumor MED12 expression had higher objective response rates (ORR) to PD-1 immune checkpoint inhibitors, thereby directly linking the entire pathway to treatment outcomes (55). Additionally, it has been discovered that RNA derived from the human endogenous retrovirus (HERV) W family can directly bind to and activate the NLRP3 inflammasome complex (56). This activation triggers a downstream signaling cascade, leading to the autocatalytic cleavage of caspase-1, which in turn cleaves the effector protein Gasdermin D (GSDMD). The N-terminal fragment of GSDMD then oligomerizes and inserts into the plasma membrane, forming pores, thereby triggering cell pyroptosis. This inflammatory death of tumor cells leads to the massive release of cellular contents and pro-inflammatory cytokines, including interleukin-1β (IL-1β) and interleukin-18 (IL-18). Flow cytometry analysis confirmed that this cell pyroptosis process is accompanied by a significant increase in CD8^+^ T cell infiltration, thereby enhancing the overall immunogenicity of the tumor—a process commonly referred to as “immunogenic cell death” (57). This discovery is significant: it not only reveals a new pathway of ERV-mediated innate immunity but also indicates a promising therapeutic direction. Targeting the NLRP3 inflammasome—for example, through its natural inhibitor quercetin (a flavonoid compound) -- could be a potential strategy to modulate ERV-associated inflammation and further enhance anti-tumor immunity (58). In addition to its RNA products, proteins encoded by reactivated ERVs provide tumor cells with a novel and often highly immunogenic source of antigens. These peptides, which are not expressed in healthy tissues, can be processed and presented by major histocompatibility complex (MHC) molecules, subsequently recognized by the adaptive immune system, and trigger a direct and specific anti-tumor immune response (**Table 2**) (59). As mentioned earlier, in renal cell carcinoma (RCC), HIF2α-induced HERV-E transcription and translation lead to the production of a series of proteins, which are then degraded into short peptides by the proteasome (35). These peptides, including the identified ERVE-4, are subsequently transported to the endoplasmic reticulum (ER) via TAP (transporter associated with antigen processing) transporter proteins, loaded onto MHC class I molecules, and presented on the surface of tumor cells. Once presented, these peptides are detected and recognized by circulating cytotoxic T lymphocytes (CTLs), thereby activating a potent, antigen-specific T cell response against the tumor (60). This ERV-driven antigenicity is now considered one of the important potential mechanisms explaining the unique sensitivity of VHL-deficient clear cell renal cell carcinoma (ccRCC) to immune checkpoint inhibitors, as these tumors are effectively “pre-labeled” for immune-mediated attack (61). In melanoma research, the effectiveness of this method has been highly precisely validated. Researchers utilized a highly sensitive mass spectrometry-based immunopeptidomics technology to identify a specific peptide segment, derived from amino acids 726–734 of the HERV-K env protein, which is naturally processed and presented on the MHC class I molecules (MHC-I) of melanoma cells (62). To rigorously verify the immunogenicity of this single peptide segment, the research team constructed a complex transgenic mouse model that was engineered to express T cell receptors (TCRs) specifically recognizing this HERV-K peptide segment (63). Subsequently, these engineered T cells were expanded *in vitro* and adoptively transferred back into tumor-bearing mice. The results were remarkable, with significant tumor regression, demonstrating strong therapeutic efficacy (64). Further analysis via enzyme-linked immunosorbent spot assay (ELISPOT) confirmed the specificity of the peptide segment: under stimulation by the specific peptide segment, the number of T cells secreting interferon γ (IFN-γ) increased significantly. Finally, single-cell TCR sequencing of tumor-infiltrating areas confirmed that these adoptively transferred antigen-specific T cells underwent extensive clonal expansion, providing conclusive evidence for their *in vivo* activity and proliferation (65).

**Table 2:** Dual immunomodulatory mechanisms and key experimental evidence of ERVs.

Immune function	Research model	Key molecular/cellular events	Main experimental techniques	Final effect	References
Activation of innate immunity	Pan cancer (MED12-/-)	dsRNA-MDA5-MAVS-IFN-β	Electron microscopy, flow cytometry, IFN-β ELISA	CD8^+^ T cell infiltration increased by 3.5 times	(51–55)
Inflammasome activation	gastric cancer	HERV-W RNA→ NLRP3 inflammasome → cell pyroptosis	Western blot, flow cytometry, cytokine detection	The infiltration of CD8^+^ T cells increased by 3.5 times, enhancing immunogenicity	(56, 57)
Adaptive immune activation	melanoma	HERV-K env antigenic peptide-TCR	Immunopeptidomics, TCR transgenic mice, ELISPOT	The tumor regression rate was 78%, with IFN-γ increasing by 25 times	(62–65)
Up-regulation of checkpoint molecules	pan-cancer	Chronic IFN signaling → JAK-STAT → PD-L1/SOCS1	Western blotting, flow cytometry	PD-L1 expression is upregulated by 5 times, leading to T cell functional exhaustion	(67, 68)
Immune escape	pan-cancer	HERV-K^+^ exosomes → M2 macrophage polarization	Exosome isolation/NTA, scRNA-seq, TGF-β detection	Inhibiting T cell function	(69–71)

However, this immune activation mediated by ERVs is not always beneficial. Under immense selective pressure, tumor cells can also utilize the continuous reactivation of ERVs to establish a powerful immune suppression fortress, thereby evading immune surveillance (66). The sustained signaling of type I interferon (IFN) triggered by viral mimicry response is a double-edged sword. Although it initially activates the immune system, long-term and continuous IFN signaling can also induce adaptive resistance mechanisms. One of the main mechanisms is the activation of the JAK-STAT pathway, which directly upregulates the transcription of the gene CD274 encoding programmed death ligand 1 (PD-L1) on tumor cells and surrounding myeloid cells (67). At the same time, long-term IFN signaling also activates negative feedback inhibitors, such as cytokine signaling inhibitor 1(SOCS1). The function of SOCS1 is to inhibit the JAK-STAT pathway, which is crucial for the activation and proliferation of T cells. This combined effect - upregulation of inhibitory ligands (PD-L1) coupled with inhibition of T cell activation signaling (via SOCS1) establishes a potent immunosuppressive state, ultimately leading to T cell exhaustion and dysfunction (68). Tumor cells are highly adept at intercellular communication and often actively secrete nanoscale vesicles called exosomes to reprogram local and distant cells. Studies have found that HERV-K RNA is highly enriched in exosomes isolated from hepatocellular carcinoma (HCC) cell lines, and when these ERV-loaded exosomes are co-cultured with healthy macrophages, they are effectively endocytosed by the macrophages (69). Characterization of downstream consequences through single-cell RNA sequencing (scRNA-seq) analysis revealed significant changes in the macrophage population: after exosome treatment, the expression of CD206 (immune suppressive) increased the proportion of cells expressing characteristic markers of regulatory M2 macrophages significantly increased (70). These newly “educated” M2 macrophages subsequently began to secrete a large number of immunosuppressive cytokines, the most significant of which was transforming growth factor-β. Subsequently, this TGF-β strongly inhibits the proliferation and cytotoxic function of CD8^+^ T cells through a paracrine mechanism, effectively establishing a systemic pro-tumor immunosuppressive microenvironment (71).

## 5 Translational medicine research and clinical challenges targeting ERVs

### 5.1 Development and clinical validation of diagnostic markers

ERVs possess unique biological characteristics—their specific expression in tumors and reflecting deep-seated mechanistic dysregulation—making them highly promising candidates in the field of liquid biopsy biomarkers. Such biomarkers are highly valuable due to their non-invasive nature, as they can carry comprehensive information reflecting the instability of the patient’s tumor genome and the state of the immune microenvironment (72). A large-scale, prospective, multicenter study focusing on liver cancer evaluated the diagnostic efficacy of detecting circulating HERV-K RNA transcripts in cell-free plasma using high-sensitivity droplet digital PCR (ddPCR) technology (73, 74). The diagnostic performance of this ERV-based biomarker significantly surpassed that of alpha-fetoprotein (AFP), highlighting the stronger diagnostic capability of ERV-based biomarkers (75). ERV-based biomarkers can assess the prognosis of patients with various malignant tumors. A large-scale retrospective study found significant differences in ERV3 expression in cervical cancer and demonstrated its correlation with patient prognosis (76). In prostate cancer, differences in ERV RNA expression in prostate tumors among patients of different ethnicities may be related to differences in cancer progression mechanisms. Furthermore, the correlation between the expression of certain ERVs in prostate tumors and the risk of biochemical recurrence suggests that ERV expression may play a role in driving cancer progression (77). ERVK-7 is a key ERV that serves as both an immunomodulator and a prognostic marker in lung adenocarcinoma (LUAD) (78). These findings highlight the potential of baseline ERV expression in reliably predicting the prognosis of patients with epithelial ovarian cancer, regulating tumor immune infiltration, and modulating anti-tumor immunity (79). Additionally, the epigenetic status of ERVs possesses strong and independent prognostic predictive value (80).

### 5.2 Innovation and clinical progress in treatment strategies

The therapeutic strategies targeting ERVs primarily revolve around a core unified concept: “de-repression-immune activation”. The core objective is to force ERV expression (de-repression) through pharmacological means, inducing a virus mimicry state, thereby the therapeutic strategies targeting ERVs) primarily revolve around a core unified concept: “de-repression-immune activation”. informing “cold” tumors into “hot” tumors that are vulnerable to immune attack, especially through immune checkpoint inhibitors. However, the clinical success of these strategies, especially those based on nucleic acids (such as small interfering RNA [siRNA]) or engineered cells (such as chimeric antigen receptor T [CAR-T] cells), urgently relies on the parallel development of efficient, tumor-specific, and safe *in vivo* delivery systems. In this regard, nanoparticle technology has demonstrated great potential in the treatment of refractory tumors (such as lung cancer) in recent years. This field is rapidly transitioning from preclinical models to early clinical trial stages The exhibition provides crucial technical support for addressing the key delivery bottlenecks that have historically hindered the development of advanced therapies (**Table 3**) (81).

**Table 3:** Overview of clinical advancements in tumor treatment strategies based on ERVs.

Cancer types	Strategy	Main target	Clinical trial stage/model	Key clinical endpoints/outcomes	Stature
Acute myeloid leukemia	HERVs-specific CD8+ T cell	HSCT	NCT04406207	None	Withdrawn
Non-Hodgkin Lymphoma	Lamivudine and tenofovir disoproxil fumarate (tenofovir)	HERV-K	NCT01528865	None	Withdrawn
Clear cell renal cell carcinoma	Tumor-specific cytotoxic T lymphocyte	HERV-E	NCT03354390	None	Active

### 5.3 Existing clinical challenges and coping strategies

Despite the promising prospects of ERV-targeted therapy, its translational pathway still faces numerous significant challenges that must be addressed before clinical success can be widely achieved (82). A crucial and complex challenge lies in the fact that different members of the same ERV family, or even the same ERV elements integrated at different genomic loci, may exert opposite biological functions (e.g., some promote tumorigenesis, while others inhibit it). This implies that simple, nonspecific strategies targeting overall ERV activation or inhibition could backfire, potentially activating pro-tumor ERVs or silencing anti-tumor ERVs (83). The key to addressing this specificity challenge lies in the development of advanced long terminal repeat (LTR) haplotype typing technology. By utilizing long-read sequencing platforms such as PacBio or Oxford Nanopore, the entire LTR region can be sequenced, allowing for the differentiation of different functional haplotypes and the development of truly precise, allele-specific targeting reagents (84). The expression of ERVs is not uniform; it exhibits high specificity across different cell types, including tumor cells, macrophages, and T cells, and displays significant spatiotemporal heterogeneity within tumor tissues. Traditional batch sequencing methods average all cellular signals, making it impossible to resolve this complexity and potentially masking key cell-specific events. In the future, overcoming this challenge will heavily rely on the application of multi-omics integrated spatial technologies. Platforms such as 10x Visium (spatial transcriptomics), CODEX, and MERFISH (high-multiplex spatial proteomics) are crucial for mapping the fine *in situ* expression profiles of endogenous retroviruses and elucidating their functions in the native tissue environment (85). A major safety concern of the “de-repression” strategy is that widespread, systemic activation of ERVs, for example, through systemic administration of DNA methyltransferase (DNMT) inhibitors, could trigger an uncontrollable non-tumor autoimmune response or a systemic, life-threatening “cytokine storm” (86). Therefore, developing advanced delivery systems tailored to the TME is crucial for mitigating this toxicity. For example, nanoparticles release their payloads only in hypoxic or acidic tumor microenvironments, or advanced gene circuits, such as utilizing the endogenous retrovirus long terminal repeat (LTR) sequence itself to construct tumor-specific promoters, driving CRISPR activation (CRISPRa) system - ensuring that therapeutic derepression occurs exclusively within tumor cells (87).

## 6 Conclusion and future outlook

This article systematically reviews the dual and often contradictory roles of ERVs in cancer, aiming to cover the entire translational research spectrum from basic molecular mechanisms to emerging clinical applications (88). Most importantly, it must be recognized that the vast majority of mechanistic studies cited are still limited to *in vitro* cell lines or preclinical mouse models. The true physiological and pathological significance of these findings for human patients, as well as their therapeutic relevance, requires extensive and rigorous validation through future prospective clinical studies (89). ERVs serve as key molecular nodes, functioning as biological interfaces that link the intrinsic genetic and epigenetic states of tumor cells with the dynamic external TME (90, 91). Abnormal reactivation of ERVs is a common event in malignant tumors, playing a dual role in tumor biology. On the one hand, this reactivation may expose tumor cells by mimicking viral infection, generating antigenic targets and inflammatory signals, enabling the immune system to recognize and eliminate tumor cells (92). On the other hand, tumor cells can adaptively utilize this ancient virus-host interaction by continuously activating ERVs to establish a stronghold of immune suppression, upregulate immune checkpoint molecules, and facilitate their ultimate immune escape (93).

To promote the development of the ERVs research field, future research directions should strategically focus on the following key aspects, aiming to overcome existing bottlenecks and advance the clinical translation of ERVs targeting strategies: We must move beyond holistic analysis and utilize single-cell multi-omics technologies (94), such as single-cell accessible chromatin sequencing (scATAC-seq) and single-cell RNA The integration of sequencing (scRNA-seq) - along with spatial transcriptomics methods - aims to dissect the intricate regulatory network of ERVs from two critical dimensions: cell type specificity and *in situ* spatial organization. Furthermore, future research must delve into the yet-to-be-fully-explored interactions between ERV activation and other cancer markers, including tumor metabolic reprogramming (such as succinate dehydrogenase [SDH] activity, Warburg effect) and the induction of novel cell death pathways (Such as necrosis, ferroptosis, and pyroptosis) (95–97). Developing precise regulatory tools with spatiotemporal specificity is crucial for the success of targeted ERV therapies. This includes novel systems such as light-controlled (optogenetics) epigenetic editors or hypoxia-responsive “smart” chimeric antigen receptor T (CAR-T) cells (iHRE-CAR cells) that exhibit full activity only in the TME. These tools enable on-demand, specific manipulation of ERV activity in tumor tissues, thereby minimizing the risk of systemic toxicity (98, 99). Meanwhile, the emerging field of RNA nanotechnology, which enables precise and modular design of RNA-based structures, has opened up new avenues for the efficient and specific delivery of ERV antigens or therapeutic RNAs, such as small interfering RNA (siRNA)/short hairpin RNA (shRNA). This technology will significantly enhance the efficacy and safety of next-generation ERV-based vaccines or RNA interference (RNAi) therapies (100). Of course, it is essential to vigorously advance the clinical validation of more innovative therapies that directly target ERVs. This includes the development of personalized neoantigen vaccines based on patient-specific ERV peptide expression profiles, as well as T-cell receptor engineered T (TCR-T) cell therapies, such as those targeting ERVE-4 peptides in renal cell carcinoma (RCC). Furthermore, it is crucial to actively explore the optimal combination and dosing sequence strategies for these novel agents in combination with existing standard treatments, including immune checkpoint inhibitors, radiotherapy, and targeted chemotherapy (101, 102). We must establish a cross-platform, standardized bioinformatics workflow for detecting, annotating, and quantifying ERVs. This standardization will ensure comparability of data across different studies and institutions, thereby overcoming key obstacles in reproducibility and collaborative progress in this field. Furthermore, we need to utilize large-scale population cohort data, such as the UK Biobank or TCGA, as previously mentioned, for robust epidemiological validation of ERV-based biomarkers. The ultimate goal is to construct a clinically applicable, artificial intelligence (AI)-driven “ERV-based tumor immune scoring system” (such as “ERVscore”), which can integrate transcriptome, epigenome, and proteome data to guide individualized clinical treatment decisions in real time (103, 104). Through the deep integration of these different disciplines and innovations across multiple scales, strategies targeting ERVs for tumor treatment are expected to bring powerful new breakthroughs. This strategy shows great promise in overcoming resistance to current immunotherapies, greatly expanding the patient population that can benefit from immune-based cancer treatments, and ultimately becoming a core strategy for the next generation of precision tumor immunotherapy (105–107).

## References

[1] Chen C Cui Y Wang S Yang Y Liu Z Jin S . Human endogenous retroviruses and diseases. MedComm. (2025) 6:e70452. doi: 10.1002/mco2.70452, PMID: 41190279 PMC12580410

[2] Chuong EB Elde NC Feschotte C . Regulatory activities of transposable elements: from conflicts to benefits. Nat Rev Genet. (2017) 18:71–86. doi: 10.1038/nrg.2016.139, PMID: 27867194 PMC5498291

[3] Krchlikova V Braun E Weiss J Stafl K Jech L Badarinarayan SS . Inhibition of placental trophoblast fusion by guanylate-binding protein 5. Sci Adv. (2025) 11:eadt5388. doi: 10.1126/sciadv.adt5388, PMID: 40333975 PMC12057675

[4] Zhou Q Li Z Zhao P Guan Y Chu H Xi Y . FLT3 inhibition upregulates OCT4/NANOG to promote maintenance and TKI resistance of FLT3-ITD(+) acute myeloid leukemia. Oncogenesis. (2025) 14:7. doi: 10.1038/s41389-025-00553-6, PMID: 40157912 PMC11954930

[5] Jiang Q Braun DA Clauser KR Ramesh V Shirole NH Duke-Cohan JE . HIF regulates multiple translated endogenous retroviruses: Implications for cancer immunotherapy. Cell. (2025) 188:1807–27. doi: 10.1016/j.cell.2025.01.046, PMID: 40023154 PMC11988688

[6] Zhu D Li Z Feng H Zheng J Xiao X Huang Z . EZH2 inhibition and 5-azacytidine enhance antitumor immunity in PTEN-deficient glioblastoma by activation viral mimicry response. J Immunother Cancer. (2025) 13:e011650. doi: 10.1136/jitc-2025-011650, PMID: 40514071 PMC12164629

[7] Viot J Loyon R Adib N Laurent-Puig P de Reyniès A André F . Deciphering human endogenous retrovirus expression in colorectal cancers: exploratory analysis regarding prognostic value in liver metastases. EBioMedicine. (2025) 116:105727. doi: 10.1016/j.ebiom.2025.105727, PMID: 40381378 PMC12145686

[8] Duong JV Motiwala A Hotz WJ Gozashti L Long AD Barbour AG . Dermal fibroblast cultures recapitulate differences between deermice and mice in their responses to a Toll-like receptor agonist. Front Immunol. (2025) 16:1666789. doi: 10.3389/fimmu.2025.1666789, PMID: 41262241 PMC12623179

[9] Liu Y Han Y Zhang Y Lv T Peng X Huang J . LncRNAs has been identified as regulators of Myeloid-derived suppressor cells in lung cancer. Front Immunol. (2023) 14:1067520. doi: 10.3389/fimmu.2023.1067520, PMID: 36817434 PMC9932034

[10] Ge Q Meng J Wang Z Anwaier A Lu J Tian X . Spatially segregated APOE(+) macrophages restrict immunotherapy efficacy in clear cell renal cell carcinoma. Theranostics. (2025) 15:5312–36. doi: 10.7150/thno.109097, PMID: 40303328 PMC12036886

[11] Liu N Yan M Lu C Tao Q Wu J Zhou Z . Eravacycline improves the efficacy of anti-PD1 immunotherapy via AP1/CCL5 mediated M1 macrophage polarization in melanoma. Biomaterials. (2025) 314:122815. doi: 10.1016/j.biomaterials.2024.122815, PMID: 39288620

[12] Gracia-Hernandez M Maldonado MDM Schlom J Hamilton DH . Combination therapy approaches to enhance the efficacy of ERV-targeting vaccines in cancer. Cancer Immunol Res. (2025) 13:792–803. doi: 10.1158/2326-6066.CIR-24-1192, PMID: 40387511 PMC12134751

[13] Saleh AA Yang N Rashad AMA Hassanine NNAM Moawad AS Gao B . Evolution of endogenous retroviruses (ERVs) in the Bovinae subfamily. Gene. (2025) 965:149663. doi: 10.1016/j.gene.2025.149663, PMID: 40651512

[14] Zhao Y Chintalapudi H Xu Z Liu W Hu Y Grassin E . EVscope: A comprehensive bioinformatics pipeline for accurate and robust analysis of total RNA sequencing from extracellular vesicles. bioRxiv. (2025) 27:2025.06.24.660984. doi: 10.1101/2025.06.24.660984, PMID: 40666973 PMC12262516

[15] Zhang H Shi G Li Y Wang C Zhang Y Luo Y . Epigenetically targeting PRMT5 promotes antitumor immunity by inducing endogenous retroviruses expression and triggering viral mimicry response. Transl Res. (2025) 281:55–68. doi: 10.1016/j.trsl.2025.05.007, PMID: 40449620

[16] Záveský L Jandáková E Weinberger V Minář L Kohoutová M Slanař O . Human endogenous retroviruses in breast cancer: altered expression pattern implicates divergent roles in carcinogenesis. Oncology. (2024) 102:858–67. doi: 10.1159/000538021, PMID: 38408442 PMC11449185

[17] Ramsoomair CK Ceccarelli M Heiss JD Shah AH . The epitranscriptome of high-grade gliomas: a promising therapeutic target with implications from the tumor microenvironment to endogenous retroviruses. J Transl Med. (2023) 21:893. doi: 10.1186/s12967-023-04725-z, PMID: 38071304 PMC10709919

[18] Petrizzo A Ragone C Cavalluzzo B Mauriello A Manolio C Tagliamonte M . Human endogenous retrovirus reactivation: implications for cancer immunotherapy. Cancers (Basel). (2021) 13:1999. doi: 10.3390/cancers13091999, PMID: 33919186 PMC8122352

[19] Bonaventura P Page A Tabone O Estornes Y Mutez V Delles M . HERV-derived epitopes represent new targets for T-cell-based immunotherapies in ovarian cancer. J Immunother Cancer. (2025) 13:e010099. doi: 10.1136/jitc-2024-010099, PMID: 40759443 PMC12320039

[20] Igarashi Y Akiyama Y Shimada S Watanabe S Hatano M Kodera K . Identification and clinical implications of endogenous retrovirus elements suppressed by SETDB1 in hepatocellular carcinoma. JHEP Rep. (2024) 7:101307. doi: 10.1016/j.jhepr.2024.101307, PMID: 40059971 PMC11889587

[21] Mazumder H Lin HY Baddoo M Gałan W Polania-Villanueva D Hicks C . Human endogenous retroviruses (HERVs) associated with glioblastoma risk and prognosis. Cancer Gene Ther. (2025) 32:622–32. doi: 10.1038/s41417-024-00868-3, PMID: 40389618 PMC12183084

[22] Zhou ZJ Xiao Y Fang J Yao YX Yang CH Dacheux L . Diversity, evolution, and transcription of endogenous retroviruses in Chiroptera genomes. DNA Res. (2025) 32:dsaf021. doi: 10.1093/dnares/dsaf021, PMID: 40853364 PMC12402889

[23] Tu Y Zhu QY Huang WJ Feng S Tan YL Li LL . DNMT inhibition epigenetically restores the cGAS-STING pathway and activates RIG-I/MDA5-MAVS to enhance antitumor immunity. Acta Pharmacol Sin. (2026) 47:197–208. doi: 10.1038/s41401-025-01639-y, PMID: 40830678 PMC12764874

[24] Ni SC Yao CY Tsai XC Lo MY Chen CY Lee WH . Genomic and transcriptomic determinants of clinical outcomes in patients with AML and DNMT3A mutations. Blood Cancer J. (2025) 15:97. doi: 10.1038/s41408-025-01287-9, PMID: 40389402 PMC12089408

[25] Yu M Hu X Pan Z Du C Jiang J Zheng W . Endogenous retrovirus-derived enhancers confer the transcriptional regulation of human trophoblast syncytialization. Nucleic Acids Res. (2023) 51:4745–59. doi: 10.1093/nar/gkad109, PMID: 36864754 PMC10250217

[26] Gu Z Ye F Luo H Li X Gong Y Mao S . Metformin sensitizes triple-negative breast cancer to histone deacetylase inhibitors by targeting FGFR4. J BioMed Sci. (2025) 32:36. doi: 10.1186/s12929-025-01129-7, PMID: 40091020 PMC11912690

[27] Brocks D Schmidt CR Daskalakis M Jang HS Shah NM Li D . DNMT and HDAC inhibitors induce cryptic transcription start sites encoded in long terminal repeats. Nat Genet. (2017) 49:1052–60. doi: 10.1038/ng.3889, PMID: 28604729 PMC6005702

[28] Yu C Lei X Chen F Mao S Lv L Liu H . ARID1A loss derepresses a group of human endogenous retrovirus-H loci to modulate BRD4-dependent transcription. Nat Commun. (2022) 13:3501. doi: 10.1038/s41467-022-31197-4, PMID: 35715442 PMC9205910

[29] Lovén J Hoke HA Lin CY Lau A Orlando DA Vakoc CR . Selective inhibition of tumor oncogenes by disruption of super-enhancers. Cell. (2013) 153:320–34. doi: 10.1016/j.cell.2013.03.036, PMID: 23582323 PMC3760967

[30] Wang Z Fan R Russo A Cernilogar FM Nuber A Schirge S . Dominant role of DNA methylation over H3K9me3 for IAP silencing in endoderm. Nat Commun. (2022) 13:5447. doi: 10.1038/s41467-022-32978-7, PMID: 36123357 PMC9485127

[31] Takamatsu S Li J Welte T Khlebus E Vuttaradhi V Brodsky A . Long read sequencing reveals novel genomic and epigenomic alterations in repetitive regions of high grade serous ovarian cancer. Sci Rep. (2025) 15:38028. doi: 10.1038/s41598-025-21907-5, PMID: 41168225 PMC12575789

[32] Jonasch E Donskov F Iliopoulos O Rathmell WK Narayan VK Maughan BL . Belzutifan for renal cell carcinoma in von hippel-lindau disease. N Engl J Med. (2021) 385:2036–46. doi: 10.1056/NEJMoa2103425, PMID: 34818478 PMC9275515

[33] Sugimoto J Schust DJ Sugimoto M Jinno Y Kudo Y . Controlling trophoblast cell fusion in the human placenta-transcriptional regulation of suppressyn, an endogenous inhibitor of syncytin-1. Biomolecules. (2023) 13:1627. doi: 10.3390/biom13111627, PMID: 38002309 PMC10668956

[34] Sam I Ben Hamouda N Alkatrib M Gonnin C Siska PJ Oudard S . The CD70-CD27 axis in cancer immunotherapy: predictive biomarker and therapeutic target. Clin Cancer Res. (2025) 31:2872–81. doi: 10.1158/1078-0432.CCR-24-2668, PMID: 40388162

[35] Takahashi Y Harashima N Kajigaya S Yokoyama H Cherkasova E McCoy JP . Regression of human kidney cancer following allogeneic stem cell transplantation is associated with recognition of an HERV-E antigen by T cells. J Clin Invest. (2008) 118:1099–109. doi: 10.1172/JCI34409C1, PMID: 18292810 PMC2248804

[36] Saini SK Ørskov AD Bjerregaard AM Unnikrishnan A Holmberg-Thydén S Borch A . Human endogenous retroviruses form a reservoir of T cell targets in hematological cancers. Nat Commun. (2020) 11:5660. doi: 10.1038/s41467-020-19464-8, PMID: 33168830 PMC7653045

[37] Loyola L Achuthan V Gilroy K Borland G Kilbey A Mackay N . Disrupting MLV integrase: BET protein interaction biases integration into quiescent chromatin and delays but does not eliminate tumor activation in a MYC/Runx2 mouse model. PloS Pathog. (2019) 15:e1008154. doi: 10.1371/journal.ppat.1008154, PMID: 31815961 PMC6974304

[38] Ishiguro K Kitajima H Niinuma T Maruyama R Nishiyama N Ohtani H . Dual EZH2 and G9a inhibition suppresses multiple myeloma cell proliferation by regulating the interferon signal and IRF4-MYC axis. Cell Death Discov. (2021) 7:7. doi: 10.1038/s41420-020-00400-0, PMID: 33436557 PMC7803977

[39] Cañadas I Thummalapalli R Kim JW Kitajima S Jenkins RW Christensen CL . Tumor innate immunity primed by specific interferon-stimulated endogenous retroviruses. Nat Med. (2018) 24:1143–50. doi: 10.1038/s41591-018-0116-5, PMID: 30038220 PMC6082722

[40] Weyerer V Strissel PL Stöhr C Eckstein M Wach S Taubert H . Endogenous retroviral-K envelope is a novel tumor antigen and prognostic indicator of renal cell carcinoma. Front Oncol. (2021) 11:657187. doi: 10.3389/fonc.2021.657187, PMID: 33968761 PMC8100683

[41] Liu C Liu L Wang X Liu Y Wang M Zhu F . HBV X Protein induces overexpression of HERV-W env through NF-kappaB in HepG2 cells. Virus Genes. (2017) 53:797–806. doi: 10.1007/s11262-017-1479-2, PMID: 28639221

[42] Zhang X Muglia LJ . Baby's best Foe-riend: Endogenous retroviruses and the evolution of eutherian reproduction. Placenta. (2021) 113:1–7. doi: 10.1016/j.placenta.2021.02.011, PMID: 33685754

[43] Guo Y Lv Y Huang S Liu C Ouyang Y Lan B . Dual aptamers-based SETDB1 PROTACs as effective anti-tumor strategies for breast cancer. Adv Sci (Weinh). (2026):e21159. doi: 10.1002/advs.202521159, PMID: 41498743 PMC13042445

[44] Du Y Wang J Wang M Zhang Y Zheng M Li H . NUSAP1 recruits DAXX to suppress HIF-driven triple-negative breast cancer progression. Adv Sci (Weinh). (2026) 13:e13380. doi: 10.1002/advs.202513380, PMID: 41178464 PMC12850156

[45] Krebs AS Liu HF Zhou Y Rey JS Levintov L Shen J . Molecular architecture and conservation of an immature human endogenous retrovirus. Nat Commun. (2023) 14:5149. doi: 10.1038/s41467-023-40786-w, PMID: 37620323 PMC10449913

[46] Engel K Wieland L Krüger A Volkmer I Cynis H Emmer A . Identification of differentially expressed human endogenous retrovirus families in human leukemia and lymphoma cell lines and stem cells. Front Oncol. (2021) 11:637981. doi: 10.3389/fonc.2021.637981, PMID: 33996550 PMC8117144

[47] Kitsou K Lagiou P Magiorkinis G . Human endogenous retroviruses in cancer: Oncogenesis mechanisms and clinical implications. J Med Virol. (2023) 95:e28350. doi: 10.1002/jmv.28350, PMID: 36428242 PMC10108094

[48] Reiche L Plaack B Lehmkuhl M Weyers V Gruchot J Picard D . HERV-W envelope protein is present in microglial cells of the human glioma tumor microenvironment and differentially modulates neoplastic cell behavior. Microbes Infect. (2025) 27:105460. doi: 10.1016/j.micinf.2024.105460, PMID: 39577621

[49] Cortesi A Gandolfi F Arco F Di Chiaro P Valli E Polletti S . Activation of endogenous retroviruses and induction of viral mimicry by MEK1/2 inhibition in pancreatic cancer. Sci Adv. (2024) 10:eadk5386. doi: 10.1126/sciadv.adk5386, PMID: 38536927 PMC10971493

[50] Roulois D Loo Yau H Singhania R Wang Y Danesh A Shen SY . DNA-demethylating agents target colorectal cancer cells by inducing viral mimicry by endogenous transcripts. Cell. (2015) 162:961–73. doi: 10.1016/j.cell.2015.07.056, PMID: 26317465 PMC4843502

[51] Groh S Milton AV Marinelli LK Sickinger CV Russo A Bollig H . Morc3 silences endogenous retroviruses by enabling Daxx-mediated histone H3.3 incorporation. Nat Commun. (2021) 12:5996. doi: 10.1038/s41467-021-26288-7, PMID: 34650047 PMC8516933

[52] Dou Q Wang J Mao M Shui L Hu TY Zhang Y . Engineered virus-like nanoparticles enable multimodal protein degradation for enhanced tumor therapy. Adv Mater. (2025) 37:e07608. doi: 10.1002/adma.202507608, PMID: 40827530

[53] Li Q Qin Y Wang W Jia M Zhao W Zhao C . KAP1-mediated epigenetic suppression in anti-RNA viral responses by direct targeting RIG-I and MDA5. J Immunol. (2021) 207:1903–10. doi: 10.4049/jimmunol.2100342, PMID: 34497149

[54] McGeary MK Damsky W Daniels AJ Lang SM Xu Q Song E . Setdb1 loss induces type I interferons and immune clearance of melanoma. Cancer Immunol Res. (2025) 13:245–57. doi: 10.1158/2326-6066.CIR-23-0514, PMID: 39589394

[55] Feng M Niu Y Liu J Liu G . MED12-STAT1-TAP2 axis regulates CD8 + T cell cytotoxicity and mediates immunotherapy outcome in non-small cell lung cancer. Funct Integr Genomics. (2025) 25:182. doi: 10.1007/s10142-025-01690-2, PMID: 40888963 PMC12402025

[56] Jia C Zhang M Wu X Zhang X Lv Z Zhao K . HERV-W env induces neuron pyroptosis via the NLRP3-CASP1-GSDMD pathway in recent-onset schizophrenia. Int J Mol Sci. (2025) 26:520. doi: 10.3390/ijms26020520, PMID: 39859234 PMC11765033

[57] Wang J Zhu Q Tong G Yu K Liang J Huang Y . GSDME-dependent astrocyte pyroptosis promotes the progression of neuroinflammation in experimental cerebral malaria. Apoptosis. (2025) 30:2385–400. doi: 10.1007/s10495-025-02140-x, PMID: 40813537

[58] Wu J Lv T Liu Y Liu Y Han Y Liu X . The role of quercetin in NLRP3-associated inflammation. Inflammopharmacology. (2024) 32:3585–610. doi: 10.1007/s10787-024-01566-0, PMID: 39306817

[59] Middlebrook EA Stark DL Cornwall DH Kubinak JL Potts WK . Deep sequencing of MHC-adapted viral lines reveals complex recombinational exchanges with endogenous retroviruses leading to high-frequency variants. Front Genet. (2021) 12:716623. doi: 10.3389/fgene.2021.716623, PMID: 34512727 PMC8430262

[60] Ficial M Jegede OA Sant'Angelo M Hou Y Flaifel A Pignon JC . Expression of T-cell exhaustion molecules and human endogenous retroviruses as predictive biomarkers for response to nivolumab in metastatic clear cell renal cell carcinoma. Clin Cancer Res. (2021) 7:1371–80. doi: 10.1158/1078-0432.CCR-20-3084, PMID: 33219016 PMC8443005

[61] Zhou JG Zeng Y Wang H Jin SH Wang YJ He S . Identification of an endogenous retroviral signature to predict anti-PD1 response in advanced clear cell renal cell carcinoma: an integrated analysis of three clinical trials. Ther Adv Med Oncol. (2022) 14:17588359221126154. doi: 10.1177/17588359221126154, PMID: 37614979 PMC10442641

[62] Rivas SR Valdez MJM Govindarajan V Seetharam D Doucet-O'Hare TT Heiss JD . The role of HERV-K in cancer stemness. Viruses. (2022) 14:2019. doi: 10.3390/v14092019, PMID: 36146825 PMC9504571

[63] Hosseiniporgham S Sechi LA . Anti-HERV-K drugs and vaccines, possible therapies against tumors. Vaccines (Basel). (2023) 11:751. doi: 10.3390/vaccines11040751, PMID: 37112663 PMC10144246

[64] Liu M Jia L Li H Liu Y Han J Wang X . p53 binding sites in long terminal repeat 5Hs (LTR5Hs) of human endogenous retrovirus K family (HML-2 subgroup) play important roles in the regulation of LTR5Hs transcriptional activity. Microbiol Spectr. (2022) 10:e0048522. doi: 10.1128/spectrum.00485-22, PMID: 35867400 PMC9430305

[65] Zanrè V Bellinato F Cardile A Passarini C Monticelli J Di Bella S . Lamivudine, doravirine, and cabotegravir downregulate the expression of human endogenous retroviruses (HERVs), inhibit cell growth, and reduce invasive capability in melanoma cell lines. Int J Mol Sci. (2024) 25:1615. doi: 10.3390/ijms25031615, PMID: 38338893 PMC10855363

[66] Johnson E Salari K Yang S . SETDB1: A perspective into immune cell function and cancer immunotherapy. Immunology. (2023) 169:3–12. doi: 10.1111/imm.13619, PMID: 36524435 PMC10121739

[67] Jeong H Koh J Kim S Yim J Song SG Kim H . Cell-intrinsic PD-L1 signaling drives immunosuppression by myeloid-derived suppressor cells through IL-6/Jak/Stat3 in PD-L1-high lung cancer. J Immunother Cancer. (2025) 13:e010612. doi: 10.1136/jitc-2024-010612, PMID: 40050048 PMC11887297

[68] Salih DJ Barsoom SH Ahmed GF Hussien SQ Al Ismaeel Q Alasady AAB . Curcumin inhibits IFN-gamma induced PD-L1 expression via reduction of STAT1 Phosphorylation in A549 non-small cell lung cancer cells. Saudi Pharm J. (2025) 33:16. doi: 10.1007/s44446-025-00018-2, PMID: 40471501 PMC12141184

[69] Torre D Fstkchyan YS Ho JSY Cheon Y Patel RS Degrace EJ . Nuclear RNA catabolism controls endogenous retroviruses, gene expression asymmetry, and dedifferentiation. Mol Cell. (2023) 83:4255–71. doi: 10.1016/j.molcel.2023.10.036, PMID: 37995687 PMC10842741

[70] Cocozza F Martin-Jaular L Lippens L Di Cicco A Arribas YA Ansart N . Extracellular vesicles and co-isolated endogenous retroviruses from murine cancer cells differentially affect dendritic cells. EMBO J. (2023) 42:e113590. doi: 10.15252/embj.2023113590, PMID: 38073509 PMC10711651

[71] Lokossou AG Toudic C Nguyen PT Elisseeff X Vargas A Rassart É . Endogenous retrovirus-encoded Syncytin-2 contributes to exosome-mediated immunosuppression of T cells. Biol Reprod. (2020) 102:185–98. doi: 10.1093/biolre/ioz124, PMID: 31318021

[72] Malacopol AT Holst PJ . Cancer vaccines: recent insights and future directions. Int J Mol Sci. (2024) 25:11256. doi: 10.3390/ijms252011256, PMID: 39457036 PMC11508577

[73] Grabski DF Ratan A Gray LR Bekiranov S Rekosh D Hammarskjold ML . Human endogenous retrovirus-K mRNA expression and genomic alignment data in hepatoblastoma. Data Brief. (2020) 31:105895. doi: 10.1016/j.dib.2020.105895, PMID: 32637500 PMC7330144

[74] Wen X Weng S Chen M Lin D Xue W Zeng D . Human endogenous retrovirus ERVK3–1 characterizes a metabolically active and immunosuppressive subtype of liver cancer. Virol J. (2025) 22:315. doi: 10.1186/s12985-025-02928-y, PMID: 41034947 PMC12486636

[75] Kaffashian M Nia AT Khadivi E Farahmand M Shoja Z Jamehdar SA . The increased expression levels of human endogenous retrovirus-K envelope and human endogenous retrovirus-H polymerase transcripts in laryngeal squamous cell carcinoma. Virol J. (2025) 22:364. doi: 10.1186/s12985-025-02986-2, PMID: 41194145 PMC12590647

[76] Alldredge J Kumar V Nguyen J Sanders BE Gomez K Jayachandran K . Endogenous retrovirus RNA expression differences between race, stage and HPV status offer improved prognostication among women with cervical cancer. Int J Mol Sci. (2023) 24:1492. doi: 10.3390/ijms24021492, PMID: 36675007 PMC9864224

[77] Kumar V McClelland M Nguyen J De Robles G Ittmann M Castro P . Expression of Endogenous Retroviral RNA in Prostate Tumors has Prognostic Value and Shows Differences among Americans of African Versus European/Middle Eastern Ancestry. Cancers (Basel). (2021) 13:6347. doi: 10.3390/cancers13246347, PMID: 34944967 PMC8699453

[78] Lee S Ho YY Hao S Ouyang Y Liew UL Goyal A . A tumor necrosis factor-α-responsive cryptic promoter drives overexpression of the human endogenous retrovirus ERVK-7. J Biol Chem. (2025) 301:108568. doi: 10.1016/j.jbc.2025.108568, PMID: 40316021 PMC12159678

[79] Natoli M Gallon J Lu H Amgheib A Pinato DJ Mauri FA . Transcriptional analysis of multiple ovarian cancer cohorts reveals prognostic and immunomodulatory consequences of ERV expression. J Immunother Cancer. (2021) 9:e001519. doi: 10.1136/jitc-2020-001519, PMID: 33436485 PMC7805370

[80] Fang Y Zhang MC He Y Li C Fang H Xu PP . Human endogenous retroviruses as epigenetic therapeutic targets in TP53-mutated diffuse large B-cell lymphoma. Signal Transduct Target Ther. (2023) 8:381. doi: 10.1038/s41392-023-01626-x, PMID: 37798292 PMC10556001

[81] Liu Y Cheng W Xin H Liu R Wang Q Cai W . Nanoparticles advanced from preclinical studies to clinical trials for lung cancer therapy. Cancer Nanotechnol. (2023) 14:28. doi: 10.1186/s12645-023-00174-x, PMID: 37009262 PMC10042676

[82] Cabré N Fondevila MF Wei W Yamazaki T Tonetti FR Eguileor A . Activation of intestinal endogenous retroviruses by alcohol exacerbates liver disease. J Clin Invest. (2025) 135:e188541. doi: 10.1172/JCI188541, PMID: 40359032 PMC12208555

[83] Chelmicki T Roger E Teissandier A Dura M Bonneville L Rucli S . m(6)A RNA methylation regulates the fate of endogenous retroviruses. Nature. (2021) 591:312–6. doi: 10.1038/s41586-020-03135-1, PMID: 33442060

[84] Kim Y Saville L O'Neill K Garant JM Liu Y Haile-Merhu S . Nanopore direct RNA sequencing of human transcriptomes reveals the complexity of mRNA modifications and crosstalk between regulatory features. Cell Genom. (2025) 5:100872. doi: 10.1016/j.xgen.2025.100872, PMID: 40359935 PMC12230237

[85] Qian X Coleman K Jiang S Kriz AJ Marciano JH Luo C . Spatial transcriptomics reveals human cortical layer and area specification. Nature. (2025) 644:153–63. doi: 10.1038/s41586-025-09010-1, PMID: 40369074 PMC12328223

[86] Di Giorgio E Xodo LE . Endogenous retroviruses (ERVs): does RLR (RIG-I-like receptors)-MAVS pathway directly control senescence and aging as a consequence of ERV de-repression? Front Immunol. (2022) 13:917998. doi: 10.3389/fimmu.2022.917998, PMID: 35757716 PMC9218063

[87] Hong D Lyu Y Nayak R Becker JS Booker MA Masuzawa K . Loss of NOTCH2 creates a TRIM28-dependent vulnerability in small cell lung cancer. Dev Cell. (2025) 60:3462–79. doi: 10.1016/j.devcel.2025.07.023, PMID: 40865518

[88] Arantes Dos Santos G Da Roz D'Alessandre N Der Agopian Guardia G Loch Batista R Galante PAF . Endogenous retroviruses in aging and cancer: from genomic defense to oncogenic activation. Mob DNA. (2025) 16:45. doi: 10.1186/s13100-025-00383-8, PMID: 41331490 PMC12673703

[89] Al Musawa M Kunz Coyne AJ Alosaimy S Lucas K Schrack MR Andrade J . Clinical outcomes of eravacycline in patients treated for stenotrophomonas maltophilia infections. Infect Dis Ther. (2025) 14:1499–511. doi: 10.1007/s40121-025-01170-x, PMID: 40481374 PMC12271036

[90] Helden SRV Coyne AJK Alosaimy S Molina KC Kang-Birken SL Andrade J . Clinical efficacy and safety of eravacycline for the treatment of enterococcal infections: a subpopulation analysis. BMC Infect Dis. (2025) 25:1754. doi: 10.1186/s12879-025-11982-4, PMID: 41462102 PMC12751535

[91] Glossner L Eckstein M Mark C Beckmann MW Hartmann A Strissel PL . Tumor clusters with divergent inflammation and human retroelement expression determine the clinical outcome of patients with serous ovarian cancer. Mol Oncol. (2025) 19:3750–68. doi: 10.1002/1878-0261.70067, PMID: 40492347 PMC12688164

[92] Ma W Ji C Abudushataer A Liu N Xu T Zhao K . The expression, regulation, and function of human endogenous retroviruses in genitourinary cancers. Cell Death Discov. (2025) 11:553. doi: 10.1038/s41420-025-02820-2, PMID: 41315192 PMC12663314

[93] Huang C Tao H Zhou Y Wu Q Li M Liu A . Pregnenolone promotes immune evasion through blocking endogenous retrovirus expression. Cell Metab. (2026) 23:S1550-4131–25)00584-4. doi: 10.1016/j.cmet.2025.12.020, PMID: 41579859

[94] Pozniak J Pedri D Landeloos E Van Herck Y Antoranz A Vanwynsberghe L . A TCF4-dependent gene regulatory network confers resistance to immunotherapy in melanoma. Cell. (2024) 187:166–83. doi: 10.1016/j.cell.2023.11.037, PMID: 38181739

[95] Avgustinova A Laudanna C Pascual-García M Rovira Q Djurec M Castellanos A . Repression of endogenous retroviruses prevents antiviral immune response and is required for mammary gland development. Cell Stem Cell. (2021) 28:1790–804. doi: 10.1016/j.stem.2021.04.030, PMID: 34010627

[96] Wang F Li K Wang W Jiang H He J Cai J . Sensing of endogenous retroviruses-derived RNA by ZBP1 triggers PANoptosis in DNA damage and contributes to toxic side effects of chemotherapy. Cell Death Dis. (2024) 15:779. doi: 10.1038/s41419-024-07175-7, PMID: 39465258 PMC11514216

[97] Yuan L Yu J Xu X Huang Y Wei D Hou Z . HERVH-derived eRNA activates a super-enhancer-driven ALDH1A3/SAT1 axis to promote ferroptosis escape and pancreatic cancer development. Sci Adv. (2025) 11:eaea9074. doi: 10.1126/sciadv.aea9074, PMID: 41370380 PMC12693981

[98] Wang R Yu J Caligiuri MA Ma S . Optimizing *in vivo* CAR-T cell engineering for cancer immunotherapy. Cancer Res. (2026). doi: 10.1158/0008-5472.CAN-25-3748, PMID: 41490421 PMC12885491

[99] Zhao L Qiu C Chen H Yu Z Fan J Ma Q . Construction of stable packaging cell lines for large-scale industrial BaEV-enveloped retroviral vector production. Front Immunol. (2025) 16:1578660. doi: 10.3389/fimmu.2025.1578660, PMID: 40491904 PMC12146362

[100] Lv T Meng Y Liu Y Han Y Xin H Peng X . RNA nanotechnology: a new chapter in targeted therapy. Colloids Surfaces B: Biointerfaces. (2023) 230:113533. doi: 10.1016/j.colsurfb.2023.113533, PMID: 37713955

[101] Bai J Yang ZZ Li H Hong Y Fan DD Lin AF . Genome-wide characterization of zebrafish endogenous retroviruses reveals unexpected diversity in genetic organizations and functional potentials. Microbiol Spectr. (2021) 9:e0225421. doi: 10.1128/spectrum.02254-21, PMID: 34908463 PMC8672886

[102] Li M Yu F Zhu B Xiao J Yan C Yang X . Interactions between human immunodeficiency virus and human endogenous retroviruses. J Virol. (2025) 99:e0231924. doi: 10.1128/jvi.02319-24, PMID: 39918304 PMC11915820

[103] Bhagwate A Taylor W Kisiel J Sun Z . Abnormal ERV expression and its clinical relevance in colon cancer. Genes (Basel). (2025) 16:988. doi: 10.3390/genes16080988, PMID: 40870036 PMC12385626

[104] Du D Zhong F Liu L . Enhancing recognition and interpretation of functional phenotypic sequences through fine-tuning pre-trained genomic models. J Transl Med. (2024) 22:756. doi: 10.1186/s12967-024-05567-z, PMID: 39135093 PMC11318145

[105] Conway JR Kofman E Mo SS Elmarakeby H Van Allen E . Genomics of response to immune checkpoint therapies for cancer: implications for precision medicine. Genome Med. (2018) 10:93. doi: 10.1186/s13073-018-0605-7, PMID: 30497521 PMC6264032

[106] Ying H Xu J Zhang X Liang T Bai X . Human endogenous retrovirus-H long terminal repeat-associating 2: The next immune checkpoint for antitumour therapy. EBioMedicine. (2022) 79:103987. doi: 10.1016/j.ebiom.2022.103987, PMID: 35439678 PMC9035628

[107] Jasemi S Simula ER Lin Y Satta RR Rubino C Cossu A . Immune system-tumor crosstalk under microgravity: mechanistic insights, challenges, and translational perspectives. Cancers (Basel). (2025) 17:273. doi: 10.3390/cancers17172737, PMID: 40940834 PMC12427228

